# Lipophilicity, Pharmacokinetic Properties, and Molecular Docking Study on SARS-CoV-2 Target for Betulin Triazole Derivatives with Attached 1,4-Quinone

**DOI:** 10.3390/pharmaceutics13060781

**Published:** 2021-05-23

**Authors:** Monika Kadela-Tomanek, Maria Jastrzębska, Krzysztof Marciniec, Elwira Chrobak, Ewa Bębenek, Stanisław Boryczka

**Affiliations:** 1 Department of Organic Chemistry, Faculty of Pharmaceutical Sciences in Sosnowiec, Medical University of Silesia, Katowice, 4 Jagiellońska Str., 41-200 Sosnowiec, Poland; kmarciniec@sum.edu.pl (K.M.); echrobak@sum.edu.pl (E.C.); ebebenek@sum.edu.pl (E.B.); boryczka@sum.edu.pl (S.B.); 2 Silesian Center for Education and Interdisciplinary Research, Institute of Physics, University of Silesia, 75 Pułku Piechoty 1a, 41-500 Chorzów, Poland; maria.jastrzebska@us.edu.pl

**Keywords:** 1,4-quinone, betulin, lipophilicity, molecular docking, SARS-CoV-2 proteins

## Abstract

A key parameter in the design of new active compounds is lipophilicity, which influences the solubility and permeability through membranes. Lipophilicity affects the pharmacodynamic and toxicological profiles of compounds. These parameters can be determined experimentally or by using different calculation methods. The aim of the research was to determine the lipophilicity of betulin triazole derivatives with attached 1,4-quinone using thin layer chromatography in a reverse phase system and a computer program to calculate its theoretical model. The physiochemical and pharmacokinetic properties were also determined by computer programs. For all obtained parameters, the similarity analysis and multilinear regression were determined. The analyses showed that there is a relationship between structure and properties under study. The molecular docking study showed that betulin triazole derivatives with attached 1,4-quinone could inhibit selected SARS-CoV-2 proteins. The MLR regression showed that there is a correlation between affinity scoring values (ΔG) and the physicochemical properties of the tested compounds.

## 1. Introduction

Nowadays, the search for new drugs requires the application of computational chemistry, including experimental and in silico analysis of physicochemical properties, pharmacokinetic features, ADMET analysis, and quantitative structure–activity relationship (QSAR) research [1,2]. The therapeutic potential of a drug depends on its distribution in the body. Often, substances with high biological activity show low bioavailability and high toxicity to healthy tissues. Nanocarriers, including liposomes, micelles, and polymer nanoparticles, enable the improvement of the biodistribution of substances. Incorporation of the hydrophobic core into hydrophilic nanotransporters can effectively influence the solubility of the drug, which contributes to the improvement of permeability through cell membranes [3,4].

A key parameter in the design of new active compounds is lipophilicity, which influences the solubility and permeability through membranes. Lipophilicity affects the pharmacodynamic and toxicological profiles of compounds. These parameters can be determined experimentally or by using different calculation methods. Experimental lipophilicity is usually determined by chromatographic methods, such as reversed phase thin layer chromatography (RP-TLC), normal phase thin layer chromatography (NP-TLC), or reversed phase high performance liquid chromatography (RP-HPLC). Theoretical methods are the second way to determine lipophilicity. However, the calculated value of lipophilicity depends on the algorithm employed for calculation, and for many compounds it differs from the experimental ones [5,6,7].

**Figure 1:**
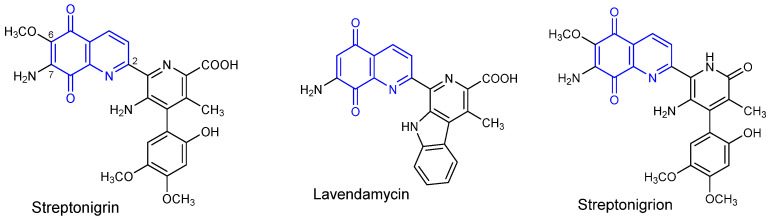
Chemical structure of 7-amino-5,8-quinolinedione antibiotics.

Natural substances, especially secondary metabolites, are often an inspiration to obtain semisynthetic compounds, which exhibit high biological activity. Microorganisms produce many secondary metabolites that affect antibacterial, antiviral, and anticancer activities. The *Streptomyces* species produce compounds containing the 5,8-quinolinedione moiety, such as *streptonigrin*, *lavendamycin*, and *streptonigron*, which exhibit a wide spectrum of biological activity (Figure 1) [8,9]. Previous studies dealt with the modification of 5,8-quinolinedione moiety at the C-2, C-6, and C-7 positions and they showed that such modification can lead to changes in biological activity [10,11,12,13,14,15,16,17,18,19].

Studies on the lipophilicity of 5,8-quinolinedione derivatives were carried out applying the RP-TLC and RP-HPLC methods. It was found that there is a relationship between lipophilicity and in silico pharmacokinetic properties of synthetic 5,8-quinolinedione derivatives [20,21,22,23]. As a continuation of our previous research, we determined the lipophilicity experimentally as well as by using computational methods for the betulin triazole derivatives with attached 1,4-quinone. The physicochemical and pharmacokinetic properties influence the bioavailability and biological activity of compounds when they are determined by computer programs. The correlation between lipophilicity and in silico determined structural parameters have been analyzed in this study.

The tested compounds contain two active moieties, i.e., 1,4-quinone and betulin. The main mechanism of activity for 1,4-quinone derivatives is the interaction with NQO1 protein, for which the overexpression is observed in many types of cancer cell lines [8,10,24]. The betulin derivatives show a wide spectrum of activities including anticancer, antiviral, antibacterial, and anti-inflammatory effects. The use of betulin and its derivatives in treatment is limited due to low bioavailability and low water solubility. For these reasons, the use of new drug nanoencapsulation procedures and/or nanoparticle-based delivery methods are a key issue. These are issues of nanomedicine interest [25,26].

Over the past decade, many betulin derivatives exhibiting high antiviral activity against human immunodeficiency virus (HIV-1), herpes simplex (HSV-1), enteric cytopathogenic human orphan virus (ECHO-6), and human cytomegalovirus (HCMV) were described [27,28,29,30,31]. The high antiviral activity of betulin derivatives has aroused interest as a possibility of using them in the treatment of COVID-19 [32,33,34,35]. The aim of the present study was to characterize the triazole betulin derivatives with attached 1,4-quinone in terms of their lipophilicity, pharmacokinetic properties, and molecular docking with SARS-CoV-2 proteins, such as Mpro and PLpro. Moreover, we examined the correlation between scoring values (ΔG) and structural properties.

## 2. Materials and Methods

### 2.1. Data Set

In this study, a series of triazole betulin derivatives with attached 1,4-quinone (**1**–**16**), as well as triazole betulin derivatives (**17**–**20**), were used. According to literature data [36], the reaction between triazole betulin derivatives (**17**–**20**) and 1,4-quinone compounds in the presence of potassium carbonate in tetrahydrofuran lead to the hybrids **1**–**16**. The chemical structures of compounds **1**–**20** are presented in Table 1.

### 2.2. Experimental Lipophilicity

The experimental lipophilicity was determined using the RP-TLC method according to the literature [20,21,37]. We used the modified silica gel as a stationary phase and (tris-hydroxymethyl)aminomethane (0.2 M, pH = 7.4) with acetone as a mobile phase. The percent of organic solvent volume was varied within the range of 60–90% in 5% increments.

The compounds **1**–**20** were dissolved in chloroform (1.0 mg/mL), then 5µL of sample solution was applied to the chromatographic plates with a micropipette. Spots were visualized by spraying with 10% ethanol solution of sulfuric acid (VI) and then heated up to 110 °C.

The obtained values of retardation factor (R_f_) were converted to R_M_ parameters according to Equation (1):

(1) $$R_{M}=\log\left(\frac{1}{R_{f}}-1\right)$$

The R_M_ parameter was calculated for every concentration of acetone and extrapolated to zero concentration of organic component in the mobile phase. The chromatographic parameter of lipophilicity (R_M0_) was calculated using Equation (2):

(2) $$R_{M}=R_{M0}+bC$$

where C is the concentration of acetone in the mobile phase, while *b* is the slope of the regression plot.

The hydrophobic index (φ_0_) was determined according to Equation (3):

(3) $$\varphi_{0}=-\frac{R_{M0}}{b}$$

### 2.3. Theoretical Lipophilicity

For compounds **1–20,** the calculated lipophilicity was determined using various online tools and free available software, including: ALOGPs, AClogP, AlogP, MLOGP, XLOGP2, XLOGP3 (German Research Center for Environmental Health, Neuherberg, Germany), milogP (Molinspiration Cheminformatics, Slovensky Grob, Slovak Republic), iLOGP, WLOGP, and SILICOS-IT (Swiss Institute of Bioinformatics, Lausanne, Switzerland) [38,39,40,41]. The pharmacokinetic and physiochemical parameters were determined using pKCMS (Bio21 Institute, Melbourne, Australia) and SwissADME software (Swiss Institute of Bioinformatics, Lausanne, Switzerland) [40,41,42,43].

### 2.4. Structure Optimization

The optimized chemical structures of compounds **1**–**16** were calculated using the DFT (B3LYP/6-311G+(d.p)) method implemented in the Gaussian 16 program package [44]. All local minima of energy were confirmed by the absence of an imaginary mode in the vibrational calculations. In our calculations, we applied the basis set of the diffuse function to heavy atoms (+) to obtain a better description of lone pair electrons with orbitals occupying a larger region of space. The obtained optimized structure is presented in Figure S1. The geometries of compounds **1**–**16** were used to determine the HOMO–LUMO energy, quantum chemical descriptor, the molecular electrostatic potential, and the molecular docking study [45]. All obtained results were visualized using the GaussView, Version 5 software package (Gaussian Inc., Wallingford, CT, USA) [46].

### 2.5. Molecular Docking Study

The three-dimensional (3D) structures (in mol2 format) of the studied compounds were generated using the ChemOffice package (version 19.1, PerkinElmer, Waltham, MA, USA) [47]. Their low-energy conformations were calculated using Gaussian 16 (revision A.03) computer code [44] at the density functional theory (DFT, B3LYP) and 6-311+G(d,p) basis sets. Calculations were performed using the X-ray coordinates of chloroquine as the input structure obtained from the Cambridge Crystallographic Data Centre (CCDC ID: CDMQUI).

Target macromolecule for molecular docking study was obtained from the Protein Data Bank (https://www.rcsb.org/, accessed on 1 April 2020). We used the 3D crystal structures of COVID-19 main protein and papain-like protease of SARS CoV-2 (PDB ID: 5R7Z and 6W9C, respectively).

Ligands and proteins used in the calculations were prepared for docking using the AutoDockTools program (Molecular Graphics Laboratory, La Jolla, CA, USA) [48]. In this study, AutoDock Vina [49] tool compiled in PyRx [50] was employed to perform molecular docking. Implementing in AutoDock Vina scoring function was mostly inspired by X-score and combined an empirical free-energy force field with a Lamarckian Genetic Algorithm. The volume was set as 25 × 25 × 25 Å. The region of interest used for AutoDock Vina docking was fixed as X = −33,784, Y = 20,933, Z = 33,306 for papain-like protease and X = −11,631, Y = 2201, Z = 23,194 for COVID-19 main protein. After calculations, only the nine highest-scored poses were returned as a docking result for ligand–cavity configuration. The complexes obtained in the Vina program were visualized using the BIOVIA Discovery Studio virtual environment (v.17.2.0.16349, Dassault systems, Vélizy-Villacoublay, France) [51].

### 2.6. Correlation and Cluster Analysis

Based on the experimental and theoretical values of lipophilicity and molecular descriptor values, the correlation and cluster analysis were performed. All data used for the cluster analysis were standardized and the cluster analysis was based on the Euclidean distance. The analysis was carried out using the Statistica 13.1 software (TIBCO Software Inc., Palo Alto, CA, USA).

## 3. Results and Discussion

### 3.1. Experimental and Theoretical Lipophilicity

The hybrids **1**–**16** and substrates **17**–**20** were examined under the RP-TLC method. For each compound, the parameter R_M_ was calculated from the retardation factor (R_f_) according to Equation (1). Equation (2) was used to determine the R_M0_ and *b* values and the results are presented in Table 2. The high correlation coefficients (r = 0.976–0.997) for all compounds show good correlation between acetone concentration and R_M_ value.

The calibration curve is required for the conversion of R_M0_ to logP_TLC_. The following compounds were used as standard substances: acetanilide, prednisone, 4-bromoacetophenone, benzophenone, anthracene, dibenzyl, 9-phenylanthracene, and dichlorodiphenyltrichloroethane (DDT), for which the literature values of logP_lit_ are in the range 1.21–6.38 [52,53]. The R_M0_ for the standard substance was determined in the same conditions as for compounds **1**–**20** (Table 3).

The calibration curve Equation (4) was determined by linear correlation between the logP_lit_ and R_M_.

logP_TLC_ = 1.139 R_M0_ + 0.561 (r = 0.996; SD = 0.188) (4)

For the standard substances the logP_TLC_ was calculated according to the calibration curve (Equation (4)). Figure S2 shows a good agreement between experimental and literature values of lipophilicity (r = 0.996).

**Figure 2:**
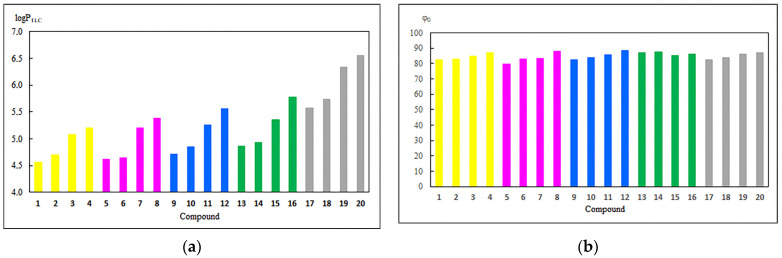
The experimental values of (**a**) lipophilicity (logP_TLC_) and (**b**) hydrophobic index (φ_0_) for 5,8-quinolinedione **1**–**4** (yellow), 5,8-isoquinoline **5**–**8** (violet), 2-methylo-5,8-quinolinedione **9**–**12** (blue), 1,4-naphthoquinone **13**–**16** (green) derivatives, and substrate **17**–**20** (grey).

Equations (3) and (4) were used to determine the logP_TLC_ and the hydrophobic index (φ_0_), respectively. The results are presented in Figure 2 and Table S1.

For compounds **1–20,** lipophilicity is in the range of 4.56–6.55. For a series of compounds **17**–**20**, the lipophilicity depends on the type of group at the C-3 position of the betulin moiety. Derivatives with hydroxyl (**17**) or oxo (**18**) groups at this position exhibit comparable values of logP_TLC_. Replacement of the hydroxyl group by acyl substituents (**19** and **20**) causes an increase in lipophilicity.

Comparing the logP_TLC_ for hybrids **1**–**16** and derivatives **17**–**20** shows that introduction of the 1,4-quinone fragment to the betulin moiety reduces the lipophilicity. The lipophilicity depends on the type of 1,4-quinone moiety and the order is as follows: 5,8-quinolinedione (**1**–**4**) > 5,8-isoquinolinedione (**5**–**8**) > 2-methyl-5,8-quinolinedione (**9**–**12**) > 1,4-naphthoquinone (**13**–**16**).

Comparing the lipophilicity of compounds **1**–**16** shows that the type of substituent at the C-3 position of betulin moiety influences the lipophilicity, and the order is as follows: hydroxy (**1**, **5**, **9**, and **13**) > oxo (**2**, **6**, **10**, and **14**) > ethanoyl (**3**, **7**, **11**, and **15**) > propanoyl (**4**, **8**, **12**, and **16**).

The hydrophobic indexes for hybrids **1**–**16** and compounds **17**–**20** are in the ranges of 79.81–88.21 and 82.62–87.13, respectively. A higher value of φ_0_ means that compounds **1**–**20** are less soluble in water. Comparing the hydrophobicity of derivatives **1**–**16** and **17**–**20** shows that the introduction of 1,4-quinone moiety slightly affects the solubility in water. In the series of **1–16,** no relationship between the type of 1,4-qinone moiety and hydrophobicity index has been observed.

**Figure 3:**
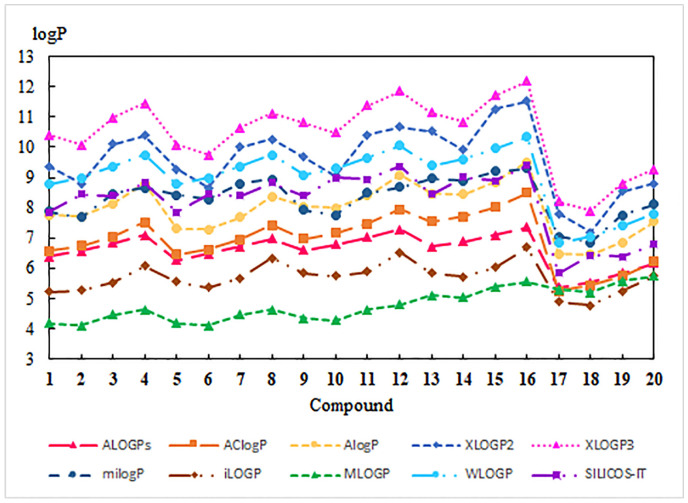
The profile of changes for theoretical lipophilicity for compounds **1**–**20**.

Theoretical values of lipophilicity were determined with the available programs [38,39,40,41]. The calculated values of logP cover a wide range from 4.11 to 12.19 depending on the mathematical module used by programs. The theoretical results are presented in Figure 3 and Table S2.

For **1**–**20**, all programs show that lipophilicity depends on the type of substituent at the C-3 position of the betulin moiety, and this correlation is consistent with the experimental results. As shown in Figure 3, compounds **17**–**20** have lower lipophilicity than hybrids **1**–**16**, which is not in agreement with the experimental results. The exception is the MLOGP program, for which the theoretical lipophilicity for derivatives **17**–**20** are similar to the experimental values (Table S2). Comparing the logP values for **1**–**16** shows that the theoretical lipophilicity slightly depends on the type of 1,4-quinone moiety. Moreover, hybrids with 5,8-quinolinedione (**1**–**4**) and 5,8-isoquinolinedione (**5**–**8**) moiety have the same molecular formula. In this case, atomistic (WLOGP) and topological (MOLGP and SILCOS-IT) methods exhibit the same value of lipophilicity, but the LogP_TLC_ is different. For this reason, to better predict the lipophilicity, it is important to find a correlation between its experimental and theoretical values. Due to the structural differences of hybrids **1**–**16** and betulin derivatives **17**–**20**, separate equations were developed for these two groups of compounds (Table 4).

**Figure 4:**
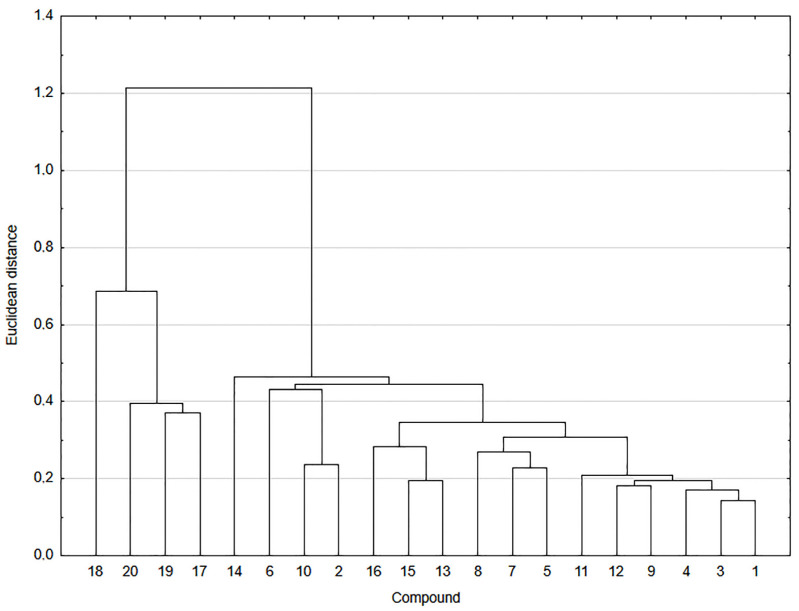
Similarity analysis of the experimental and theoretical values of lipophilicity for compounds **1**–**20**.

The relationship between the lipophilicity and structure of compounds **1**–**20** was analyzed by cluster analysis (Figure 4).

As shown in Figure 4, compounds **1**–**20** are arranged in two main clusters. The first cluster consists of betulin derivatives **17**–**20** and the second one of hybrids **1**–**16**. The high value of Euclidean distance between two clusters suggests that there is a low correlation between structures of these two groups of compounds.

The second cluster can be divided into four subclusters. In the first subcluster, the hybrids are arranged according to the type of betulin moiety, which means that they have oxo group at the C-3 position of the betulin moiety. The second and third subclusters consist of 1,4-naphthoquinone (**13**, **15**, and **16**) and 5,8-quinolinedione moiety (**5**, **7**, and **8**), respectively. Compounds containing the 5,8-quinolinedione (**1**, **3**, and **4**) and 2-methyl-5,8-quinolinedione (**9**, **11**, and **12**) are included into the subcluster four. The similarity analysis shows a strong correlation between lipophilicity and structure of hybrids **1**–**16**.

### 3.2. Physicochemical and Pharmacokinetic Properties

In the early drug discovery process, one of the important stages is to optimize the physicochemical properties of a potential drug. Based on the observation of the physicochemical properties of oral drugs, Lipinski formulated the Rule of Five. According to this rule, the lipophilicity (logP), molecular weight (MW), number of acceptors (HA), and donors (HD) of hydrogen bond should be a multiple of five [54]. In an attempt to improve the prediction of bioavailability, the rules were expanded by Veber to the number of rotatable bonds (RB) and topological polar surface area (TPSA) are also determined [55,56]. The molecular descriptors for tested hybrids **1**–**16** are presented in Table 5.

The tested hybrids **1**–**16** have a molecular mass above 500 g/mol, which means they do not meet the mass criterion. All compounds have less than five hydrogen bond donors (HD = 0–1). The 5,8-quinolinedione and 5,8-isoquinolinedione derivatives with hydroxyl and oxo groups at C-3 position of betulin (**1**–**2**, **5**–**6**, and **9**–**10**) have less than 10 hydrogen bond acceptors (HA = 9–10) and the logP_TLC_ less than 5, which means that these compounds meet three of four Lipinski rules. Only compounds with propanoyl substituents at the C-3 position of the betulin moiety (**4**, **8**, **12**, and **16**) do not meet the Veber’s rules concerning the number of rotatable bonds (RB < 10). The TPSA for **1**–**16** is less than 140 Å, which determines the oral bioavailability.

**Figure 5:**
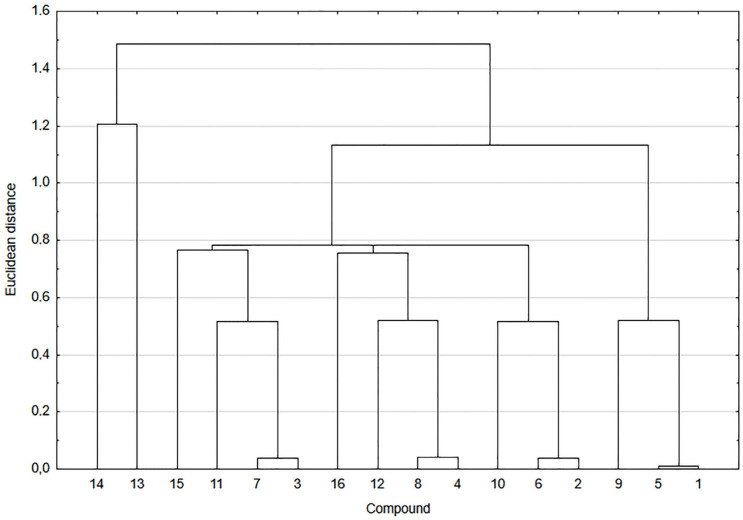
Similarity analysis of the physicochemical parameters and the experimental lipophilicity for compounds **1**–**16**.

For compounds **1**–**16**, the relationship between the type of 1,4-quinone moiety, the physicochemical parameters and experimental lipophilicity was analyzed by means of a dendrogram (Figure 5).

The similarity analysis shows two main clusters. The first cluster consists of 1,4-naphthoquinone derivatives (**13** and **14**) and the second one is divided into four subclusters. Analysis of subclusters show that compounds with the 5,8-quinolinedione (**1**–**4**) and 2-methyl-5,8-quinolinedione (**5**–**8**) exhibit similar properties. Comparing the properties of 5,8-quinolinedione (**1**–**4**) and 5,8-isoquinolinedione (**9**–**11**) shows that the position of nitrogen atom in heterocyclic ring influences their physicochemical parameters. The similarity analysis shows that nitrogen atom significantly affects properties of the tested derivatives.

The multilinear regression (MLR) Equation (5) expresses the relationship between the experimental lipophilicity (logP_TLC_) and physicochemical parameters, such as molecular mass (MW), topological polar surface (TPSA), and number of rotatable bonds (RB).

logP_TLC_ = 0.992MW − 0.488TPSA + 0.213RB − 3.362 (r = 0.985, r^2^ = 0.969, SD = 1.11, VIF = 3.12, *F* = 126.7) (5)

The correlation coefficient shows good agreement between physicochemical parameters and experimental lipophilicity. A comparison of the experimental and calculated parameters for the investigated compounds are shown in Table S3. The result shows that lipophilicity could be determined by in silico parameters.

The physicochemical properties were used to in silico calculate the pharmacokinetic parameters, which determine the absorption of the potential drug [57]. The prediction of oral and transdermal absorption was performed in silico using the Caco-2 cell (logPapp), human intestinal absorption (HIA), and skin permeability (logKp) models. Moreover, the neurotoxicity of the compounds can be designated by blood–brain barrier permeability (logBB) and central nervous system (logPS) penetration [12,58,59,60]. The pharmacokinetic parameters of compounds **1**–**16** have been determined in silico by pkCSM software and they are presented in Table 6.

According to the model used in the pkCMS software, if logPapp is more than 0.9, it means that the compound expresses the high Caco-2 permeability [42]. For the tested hybrids **1–12,** the logPapp value is in the range 0.200–0.672, which means moderate Caco-2 permeability. In the series of 5,8-quinolinedione hybrids (**1**–**12**), compounds with hydroxyl or oxo groups at the C-3 position of betulin moiety (**1**–**2**, **5**–**6**, **9**–**10**) have higher values of logPapp than hybrids with acyl group at this position (**3**–**4**, **7**–**8**, **11**–**12**). In the group of 1,4-naphthoquinone compounds (**13**–**16**), the Caco-2 permeability does not depend on the type of substituent at the C-3 position of the betulin moiety. Comparing the logPapp values shows that the permeability depends on the type of 1,4-quinone moiety and the following order is fulfilled: 1,4-naphthoquinone > 5,8-quinolinedione > 2-metyl-5,8-quionolinedione > 5,8-isoquinolnedione. For **1**–**16** the human intestinal absorption (HIA) are in the range of (97.226–98.510), which are considered to be high. The HIA index slightly depends on the type of 1,4-quinonemoiety. Hybrids **1**–**16** have a moderate skin permeability because they have the logKp lower than −2.5.

For **1**–**16**, the logBB are lower than −1, which means these hybrids poorly cross the brain–blood barrier. Compounds do not pass into the central nervous system when the logPS is less than −2. This result shows that the tested derivatives **1**–**16** are not neurotoxic.

The similarity analysis showed no correlation between pharmacokinetic parameters and the structure of compounds **1**–**16** (Figure S3). However, the cluster analysis showed that only betulin moiety influences the pharmacokinetic parameters of hybrids.

### 3.3. Molecular Properties

The energy of HOMO and LUMO orbitals determines the ability of the molecule to donated or receive an electron. The energy of orbitals can be used to calculate the global reactivity descriptors, such as: ionization potential (I), electron affinity (A), hardness (η), chemical potential (µ), electronegativity (χ), and electrophilicity index (ω) [61].

The HOMO and LUMO orbitals of **1**–**16** are presented in Figure S4. Table 7 shows the HOMO and LUMO energy, the energy gap (ΔE = E_HOMO_ − E_LUMO_), the global reactivity descriptors, and dipole moment (DM).

For all compounds **1**–**16**, the LUMO orbitals are mainly delocalized at the 1,4-quinone moiety. Localization of HOMO orbitals depends on the type of substituent at the C-3 position of the betulin moiety. The HOMO orbitals of hybrids with hydroxy and acyl groups (**1**, **3**–**5**, **7**–**9**, **11**–**13**, and **15**–**16**) at the C-3 position are mainly delocalized at the isopropenyl group and A ring of betulin. For hybrids with the oxo group (**2**, **6**, **10**, and **14**), these orbitals are delocalized at the D ring and oxo group (Figure S4). The molecular properties influence the reactivity and stability of molecules. The high value of E_HOMO_ and low value of E_LUMO_ show that the compounds are highly reactive with nucleophilic molecules. The energy gap ΔE depends on the van der Waals interaction between molecules and its value could be correlated with the biological activity of the compounds [62,63]. The hybrids **1**, **5**, **9**, and **13** are characterized by the lowest chemical hardness and the highest negative value of chemical potential, which means they are comparatively soft with high polarizability compared with other compounds in this series.

**Figure 6:**
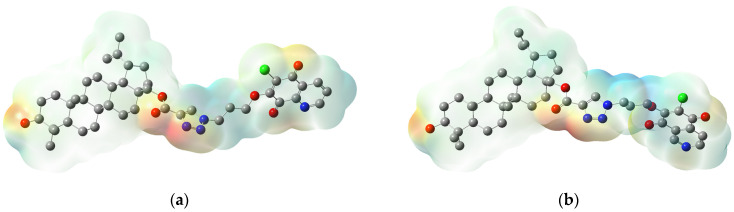

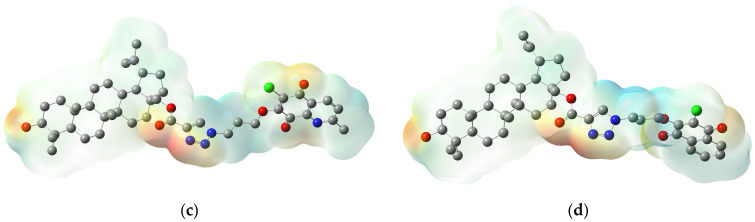
Molecular electrostatic potential plotted for hybrids: (**a**) **1**, (**b**) **5**, (**c**) **9,** and (**d**) **13**.

The arrangement of nucleophilic and electrophilic regions of a molecule influences its interaction with a biological target through hydrogen bonds and hydrophobic interactions. The distribution of the positive and negative charges of molecule is described by the molecular electrostatic potential map (MEP) [64,65]. The different charges are represented by different colors, which means that the red, blue, and green areas are negative, positive, or neutral, respectively [66]. The MEP’s are sketched for an order of (−47,935) to 47,935 kcal/mol. The maps for hybrids **1**, **5**, **9**, and **13** present in Figure 6, while for the rest of the compounds, they are shown in Figure S5. 

For all hybrids **1**–**16**, the nucleophilic regions (red color) are localized in four main area. The first and second areas are localized on 1,4-quinone moiety. The first area contains the carbonyl atom at C-5 and the second area the carbonyl group at C-8, and the nitrogen atom. The third area includes the triazole linker, and the fourth, the substituent at the C-3 position of the betulin moiety. The electrophilic regions (blue color) are localized near the methine group at 1,4-quinone moiety and the methylene group at the triazole linker, while the betulin regions are neutral (Figure 6 and Figure S5).

In each area, the local minima have been determined for **1–16,** and they are collected in Table S4. The analysis of the local minima in the first, third, and fourth areas show that the arrangement of charges does not depend on the type of 1,4-quinone moiety. However, in the second area, the amount of potential minima depends on the type of 1,4-quinone moiety, i.e., the hybrids with 5,8-quinolinedione or 5,8-isoquinolinedione moiety have two potential minima, but compounds with 1,4-naphthoquinone moiety have only one potential minima in this area (Table S4).

### 3.4. Molecular Docking Study

**Figure 7:**
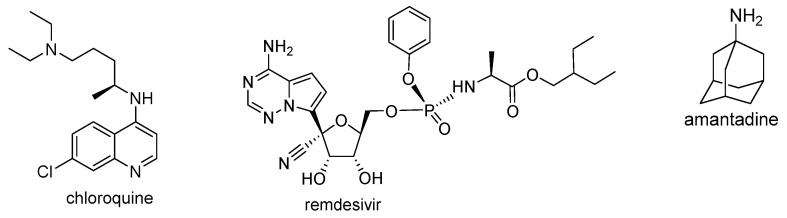
Chemical structure of drugs used in COVID-19 treatment.

Since the end of 2019, coronavirus disease 2019 (COVID-19), caused by SARS-CoV-2, has infected many people around the word. The World Health Organization (WHO) estimated that up to April 2021 more than 142 million people tested positive for COVID-19 and more than 3 million people died from this virus [67]. At the beginning of the pandemic, attempts were made to treat with chloroquine, but this treatment was halted and now chloroquine is not recommended (Figure 6) [68]. Contemporary reports indicate the effective use of amantadine and remdesivir in the treatment of patients with SARS-CoV-2 (Figure 7) [69,70].

Continuing our research on molecular docking to SARS-CoV-2 targets, we examined the interaction between hybrids **1**–**16** and Mpro and PLpro proteins using the AutoDock Vina program (referred to as Vina). As reference ligands, chloroquine, amantadine, and remdesivir were used (Figure 7).

The results obtained with the use of the Vina program are presented in Table 8. The lower the ΔG energy, the better the affinity of the tested ligands for the receptor. Calculations of the K_i_ from the binding energy of the pose generated by Vina were performed using Equations (6) and (7).

(6) $$\frac{\Delta G}{R\cdot T}{=\mathrm{lnK}}_{i}(\mathrm{for}\;T=298\;K\;\mathrm{and}\;R=1.987\;[\frac{\mathrm{kcal}}{K\cdot \mathrm{mol}}]),$$

(7) $$K_{i}=\exp(\frac{\Delta G}{R\cdot T}).$$

Based on the obtained results, it can be concluded that the tested derivatives show docking scores in the range of −9.3 to −7.8 and −8.3 to −6.4 for Mpro and PLpro, respectively. The obtained ΔG are lower compared to the reference compounds (−7.4 to −4.5 and −5.7 to −4.1 for Mpro and PLpro, respectively). This indicates that in preliminary in silico studies, hybrids **1**–**16** show a higher affinity for the proteins used than the reference drugs. Comparing the score values of hybrids **1**–**16** with betulin-5,8-quinolinedione derivatives [18] showed that the introduction of triazole linker between betulin and 1,4-quinone increased the ΔG value for Mpro and PL pro proteins.

The main protease Mpro, also known as 3CLpro, is one of the coronavirus nonstructural proteins (Nsp5). Inhibition of Mpro would prevent the virus from replication and as a consequence, Mpro is one of the coronavirus proteins designated as potential targets for drug development. According to the crystallographic data, amino acids His41, Met49, and residues 164–168 of the long strand play an important role in stabilizing the ligand-Mpro complexes [71].

**Figure 8:**
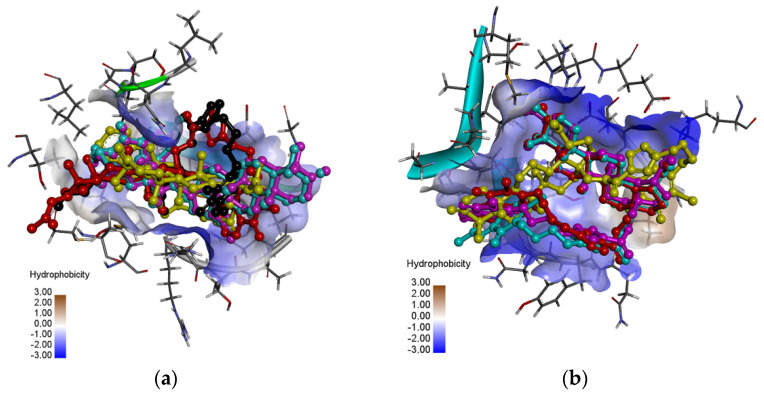
The superposition of docked ligands: **2** (violet), **6** (yellow), **10** (blue), **11** (red), and **14** (black) in the binding site of (**a**) Mpro and (**b**) PLpro.

As seen in Figure 8a, the hybrids are localized in the hydrophobic matrix of the protein, but the arrangement depends on the type of the 1,4-quinone moiety. Comparing the score values for Mpro protein shows that in the series of 5,8-quinolinedione (**1**–**4**) and 5,8-isoquinolinedione (**5**–**8**), the highest ΔG values exhibit derivatives with oxo group at the C-3 position of betulin moiety. In the group of 2-methyl-5,8-quinolinedione, better docking scores are obtained for hybrids **10** and **11**. However, in the series of 1,4-naphthoquinone derivatives (**13**–**16**), the group at the C-3 position of the betulin moiety does not influence the ΔG.

**Figure 9:**
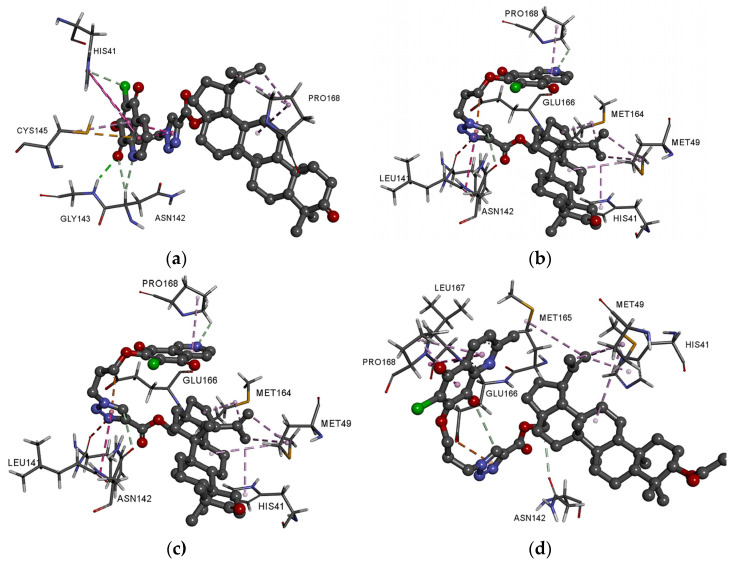

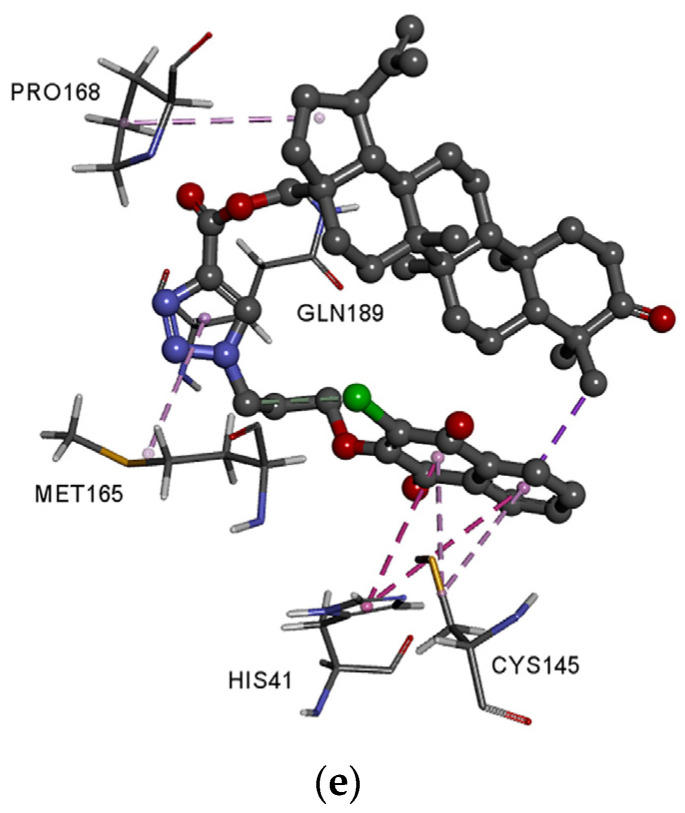
Docking pose of COVID-19 Mpro protein complex with hybrids (**a**) **2**, (**b**) **6**, (**c**) **10**, (**d**) **11**, and (**e**) **14**.

Ligands **2**, **6**, and **14**, which have the best score values, contain the same betulin moiety but different 1,4-quinone fragments. In the group of 2-methyl-5,8-quinolinedione hybrids, the lowest ΔG has hybrid **11**. For detailed analysis of molecular docking, complexes of the Mpro with **2**, **6**, **10**, **11**, and **14** ligands have been chosen. Complete models of the possible interaction in 3D and 2D views are presented in Figure 9a,e and Figure S6A–E. The detailed data about the type and length of the binding interactions between these ligands and the protein residues are summarized in Table S5.

Comparing the arrangement of hybrids **2**, **6**, **10** in the active site of the protein shows that the position of nitrogen atoms in the 5,8-quinolinedione moiety influences the interaction between the ligand and the hydrophobic matrix of protein. As seen in Figure 9a,c and Table S5, ligands **2** and **10** create the hydrogen bond and hydrophobic interaction between 1,4-quinone moiety and His41, Asn142, Gly143, and Cys145. In these complexes, the betulin moiety is stabilized by hydrophobic interaction with Pro168. Changing the 5,8-quinolinedione ring to 5,8-isoquinolinedione moiety causes different arrangement of the ligand in the hydrophobic matrix. In complex Mpro-hybrid **6**, the nitrogen atom and triazole ring create the hydrogen bond and hydrophobic interaction with Pro168 and Asn142, Glu166, Leu141, respectively (Figure 9b and Figure S7B). In this case, betulin is bound to Met49, Met165, and His41 by hydrophobic interaction. The 1,4-naphthoquione moiety (hybrid **14**) interacts with His41, Cys145, Met165, and Gln189 by hydrophobic interaction. Moreover, the triazole unit and betulin moiety are stabilized by hydrophobic interaction with Cys145 and Pro168, respectively (Figure 9e and Figure S6E).

Figure 9d and Figure S6D present the possible interaction of the best fitted compound **11** inside the binding pocket of Mpro. Corresponding amino acids that are significantly involved in the hydrophobic interactions between ligand and the betulin unit are as follows: His41, Met49, and Met165. Moreover, the whole complex is additionally stabilized by the hydrophobic interactions of the newly introduced substituents: the triazole unit with Glu166 and the 1,4-quinone moiety with Leu167 and Pro168.

It should be emphasized that the 1,4-quinone moiety plays an important role in the stabilization of Mpro hybrid complexes.

One of the attractive antiviral drug targets is the SARS-CoV-2 papain-like protease PLpro which is responsible for processing three cleavage sites of the viral polyprotein to release mature nonstructural proteins 1, 2, and 3. In the case of PLpro, the drug molecules bind to S3/S4 domains. The S3/S4 pocket contains the residues Asp164, Val165, Arg166, Glu167, Met208, Ala246, Pro247, Pro248, Tyr264, Gly266, Asn267, Tyr268, Gln269, Cys217, Gly271, Tyr273, Thr301, and Asp302 [72].

As seen in Figure 8b, the hybrids are localized in the hydrophobic matrix of the protein, but the type of 1,4-quinone slightly influences the arrangement of the hybrid in the pocket site of proteins. Comparing the score values for PLpro protein shows that in the group of 5,8-quinolinedione hybrids (**1**–**4** and **9**–**12**) the highest values have derivatives with hydroxy group at the C-3 position of betulin moiety, while in the series of **5**–**8** and **13**–**16** the best results are obtained for hybrids with the oxo group at this position.

**Figure 10:**
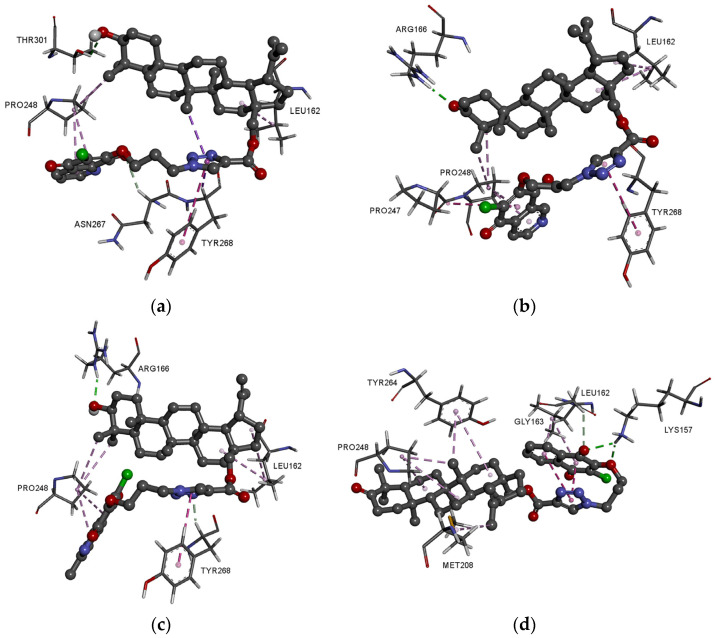
Docking pose of COVID-19 PLpro protein complex with hybrids (**a**) **1**, (**b**) **6**, (**c**) **9**, and (**d**) **14**.

In each group of 1,4-quinone, we chose one of the best ΔG, and performed detailed analysis for it. Complete models of the possible interaction in 3D and 2D views are presented in Figure 10a–d and Figure S7A–D. The detailed data about the type and length of the binding interactions between these ligands and the protein residues are summarized in Table S5.

Comparing the optimal docking post of compounds **1**, **6**, and **9** shows a general trend that the betulin moiety is bound to Pro248 and Lue162 by hydrophobic interaction (Figure 10a–c and Figure S7A–C). The hydroxyl or carbonyl group at the C-3 position creates a hydrogen bond with Thr301 (**1**) or Arg166 (**6** and **9**). The 1,4-quinone moiety and triazole ring are involved in hydrophobic interaction with Pro248 and Tyr268, respectively.

Hybrid with 1,4-naphtoquinone **14** has a different arrangement in the active site of PLpro than these with 5,8-quinolinedione or 5,8-isoquinolinedione ligands (Figure 10d and Figure S7D). The 1,4-quinone moiety creates two hydrogen bonds with Lys157 and one with Gly163. The complex is stabilized by hydrophobic interaction with Leu162, Pro248, Met208, and Tyr264. In this case, the triazole ring does not interact with the hydrophobic matrix of enzyme.

As indicated by literature date, the molecular, physicochemical, and pharmacokinetic properties can be used to determine the biological activity of the compounds [73,74]. The MLR analysis allows to find correlation between score values (∆G), experimental lipophilicity and in silico determined parameters. The MLR equation (8) shows the correlation between the score value for Mpro, experimental lipophilicity (logP_TLC_), number of rotatable bonds (RB), and the energy of LUMO (E_LUMO_) orbitals. The ∆G for PLpro correlates with molecular mass (M), number of acceptors of hydrogen bond (HA), and energy of HOMO (E_HOMO_) orbitals (Equation (9)).

ΔG_Mpro_ = −0.65 logP_TLC_ + 1.04 RB + 0.624 E_LUMO_ − 3.052 (r = 0.788, r^2^ = 0.621, SD = 0.281, *F* = 6.559, *p* = 0.007) (8)

ΔG_PLpro_ = 0.280 M − 0.57 HA + 0.38 E_HOMO_ − 30.306 (r = 0.844, r^2^ = 0.712, SD = 0.135, *F* = 9.894, *p* = 0.001) (9)

The obtained results show that the physicochemical and molecular properties of tested hybrids influence their interaction with the SARS-CoV-2 proteins.

## 4. Conclusions

In the presented study, the lipophilicity of betulin triazole derivatives with attached 1,4-quinone moiety was determined and analyzed in terms of structure, physicochemical, and pharmacokinetic parameters. Comparing the lipophilicity of betulin triazole derivatives and hybrids with attached 1,4-quinone, the introduction of 1,4-quinone moiety reduces the lipophilicity. The cluster analysis showed a correlation between the molecular structure of hybrids and their experimental and calculated lipophilicity that can be used to predict these values when designing new compounds. The bioavailability of the tested compound was described by pharmacokinetic and physicochemical properties using the Lipinski and Veber rules. The obtained in silico parameters showed that most of the hybrids could be applied orally and they did not exhibit the neurotoxic activity.

For compounds with attached 1,4-quinone, the LUMO orbitals were mainly delocalized at the 1,4-quinone moiety. Localization of HOMO orbitals depended on the type of substituent at the C-3 position of the betulin moiety. The molecular properties depended on the type of 1,4-quinone moiety. The molecular electrostatic potential showed that the negative potential sites are present in the nucleophilic atoms, i.e., nitrogen and oxygen atoms. The arrangement of the charge depends on the type of 1,4-quinone moiety.

The molecular docking study showed that betulin triazole derivatives with attached 1,4-quinone can inhibit selected SARS-CoV-2 proteins. The high affinity score values of Mpro and PLpro proteins resulted from the introduction of the triazole ring as a linker connecting the betulin moiety with the 1,4-quinone fragment. The multilinear regression equation showed the correlation between the score values for Mpro and PLpro proteins, and experimental lipophilicity (logP_TLC_), molecular descriptors, and global properties. The study showed that the determination of these parameters allows for the prediction of the interactions between the ligand and SARS-CoV-2 proteins.

## Figures and Tables

**Table 1:** Chemical structure and biological activity of compounds **1**–**20**.

Compound	Chemical Structure	Compound	Chemical Structure
**1**	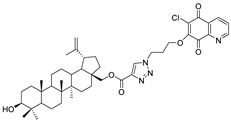	**2**	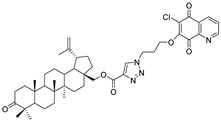
**3**	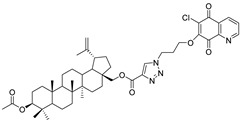	**4**	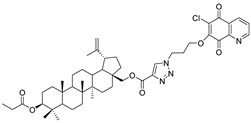
**5**	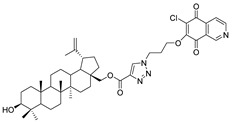	**6**	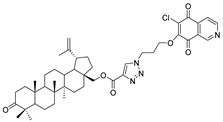
**7**	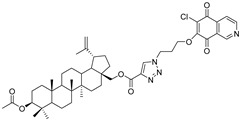	**8**	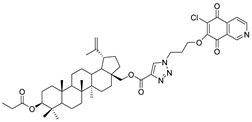
**9**	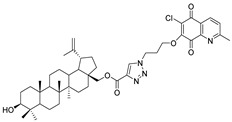	**10**	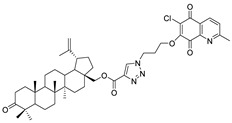
**11**	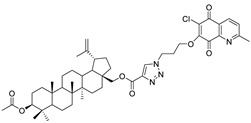	**12**	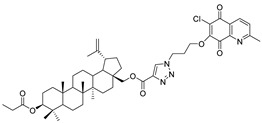
**13**	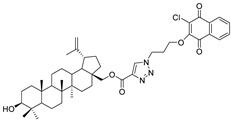	**14**	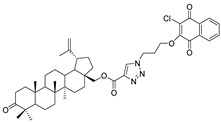
**15**	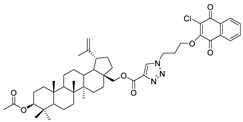	**16**	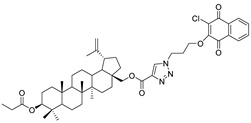
**17**	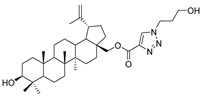	**18**	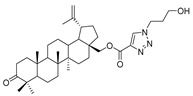
**19**	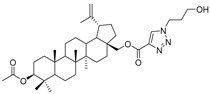	**20**	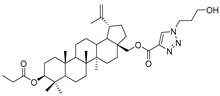

**Table 2:** The experimental values of lipophilicity (R_M0_) for compounds **1**–**20**.

Compound	R_M0_	*b*	r	SD
**1**	3.51	−0.04	0.995	0.051
**2**	3.64	−0.04	0.992	0.067
**3**	3.97	−0.05	0.996	0.047
**4**	4.08	−0.05	0.992	0.072
**5**	3.56	−0.04	0.983	0.100
**6**	3.59	−0.04	0.997	0.041
**7**	4.07	−0.05	0.985	0.101
**8**	4.24	−0.05	0.993	0.065
**9**	3.65	−0.04	0.996	0.047
**10**	3.77	−0.04	0.996	0.048
**11**	4.12	−0.05	0.995	0.054
**12**	4.39	−0.05	0.981	0.115
**13**	3.78	−0.04	0.998	0.028
**14**	3.84	−0.04	0.992	0.067
**15**	4.21	−0.05	0.979	0.120
**16**	4.58	−0.05	0.960	0.183
**17**	4.40	−0.05	0.995	0.065
**18**	4.54	−0.05	0.989	0.098
**19**	5.07	−0.06	0.995	0.073
**20**	5.26	−0.06	0.976	0.161

*b* is the slope, r is the correlation coefficient, and SD is the standard deviation for the linear relationship R_M_ = R_M0_ + *b*C.

**Table 3:** The experimental (R_M0_) and literature (logP_lit_) lipophilicity values of standard substance.

Compound	logP_lit_	R_M0_	*b*	r	SD	logP_TLC_
Acetanilide	1.21	0.55	−0.01	0.954	0.051	1.19
Prednisone	1.63	0.73	−0.02	0.932	0.077	1.39
4-Bromoacetophenone	2.43	1.89	−0.03	0.993	0.037	2.72
Benzophenone	3.18	2.37	−0.03	0.993	0.046	3.26
Anthracene	4.45	3.48	−0.04	0.994	0.053	4.52
Dibenzyl	4.79	3.65	−0.04	0.997	0.043	4.72
DDT	6.01	4.87	−0.06	0.985	0.041	6.17
9-Phenylanthracene	6.38	4.93	−0.06	0.993	0.080	6.11

*b* is the slope, r is the correlation coefficient and SD is the standard deviation for the linear relationship R_M_ = R_M0_ + *b*C.

**Table 4:** Correlation equations for experimental (LogP_TLC_) and theoretical (LogP_calc_) values of lipophilicity for compounds **1**–**16** and **17**–**20**.

Program	Correlation Equation	r	SD
Compounds **1**–**16**			
ALOGPs	logP_TLC_ = 1.094 logP_CALC_ − 2.416	0.932	0.137
AClogP	logP_TLC_ = 0.540 logP_CALC_ + 1.104	0.854	0.196
AlogP	logP_TLC_ = 0.492 logP_CALC_ + 0.987	0.843	0.203
XLOGP2	logP_TLC_ = 0.378 logP_CALC_ + 1.268	0.854	0.196
XLOGP3	logP_TLC_ = 0.472 logP_CALC_ − 0.121	0.887	0.174
milogP	logP_TLC_ = 0.527 logP_CALC_ + 0.549	0.729	0.258
iLOGP	logP_TLC_ = 0.758 logP_CALC_ + 0.620	0.887	0.174
WLOGP	logP_TLC_ = 0.755 logP_CALC_ − 2.091	0.953	0.114
MLOGP	logP_TLC_ = 0.582 logP_CALC_ + 2.353	0.723	0.260
SILICOS-IT	logP_TLC_ = 0.634 logP_CALC_ − 0.443	0.798	0.227
Compounds **17**–**20**			
ALOGPs	logP_TLC_ = 1.313 logP_CALC_ − 1.485	0.980	0.114
AClogP	logP_TLC_ = 1.078 logP_CALC_ − 0.058	0.962	0.157
AlogP	logP_TLC_ = 0.847 logP_CALC_ + 0.251	0.962	0.232
XLOGP2	logP_TLC_ = 0.567 logP_CALC_ + 1.462	0.880	0.273
XLOGP3	logP_TLC_ = 0.718 logP_CALC_ − 0.097	0.929	0.213
milogP	logP_TLC_ = 0.766 logP_CALC_ + 0.345	0.960	0.160
iLOGP	logP_TLC_ = 0.994 logP_CALC_ + 0.916	0.924	0.220
WLOGP	logP_TLC_ = 1.083 logP_CALC_ − 1.828	0.979	0.116
MLOGP	logP_TLC_ = 1.754 logP_CALC_ − 3.543	0.955	0.170
SILICOS-IT	logP_TLC_ = 0.965 logP_CALC_ − 0.092	0.815	0.332

**Table 5:** Molecular descriptors for hybrids **1**–**16**.

Compound	MW (g/mol)	HA	HD	RB	TPSA (Å)
**1**	787.42	10	1	9	133.50
**2**	785.41	10	0	9	130.34
**3**	829.46	11	0	10	139.57
**4**	843.49	11	0	11	139.57
**5**	787.42	10	1	9	133.50
**6**	785.41	10	0	9	130.45
**7**	829.46	11	0	10	139.57
**8**	843.39	11	0	11	139.57
**9**	801.45	10	1	9	133.50
**10**	799.43	10	0	9	130.34
**11**	843.49	11	0	10	139.57
**12**	857.51	11	0	11	139.57
**13**	786.44	9	1	9	120.61
**14**	784.42	9	0	9	117.45
**15**	828.47	10	0	10	126.68
**16**	842.50	10	0	11	126.68

**Table 6:** Pharmacokinetic parameters of hybrids **1**–**16**.

Compound	logBB	logPS	logKp	logPapp	HIA
**1**	−1.459	−2.481	−2.734	0.641	97.226
**2**	−1.581	−2.513	−2.735	0.639	98.193
**3**	−1.804	−2.442	−2.735	0.195	98.510
**4**	−1.831	−2.434	−2.735	0.215	98.358
**5**	−1.457	−2.466	−2.735	0.672	97.226
**6**	−1.579	−2.499	−2.735	0.671	98.193
**7**	−1.802	−2.428	−2.735	0.158	98.510
**8**	−1.830	−2.420	−2.735	0.178	98.358
**9**	−1.447	−2.443	−2.734	0.598	97.235
**10**	−1.569	−2.475	−2.735	0.597	98.090
**11**	−1.792	−2.404	−2.735	0.181	98.358
**12**	−1.820	−2.396	−2.735	0.200	98.231
**13**	−1.219	−2.215	−2.734	0.747	97.410
**14**	−1.341	−2.248	−2.735	0.745	97.838
**15**	−1.564	−2.177	−2.735	0.733	97.885
**16**	−1.592	−2.169	−2.735	0.728	97.879

**Table 7:** Electrostatic descriptor of hybrids **1**–**16**.

Hybrid	E_HOMO_ (kcal/mol)	E_LUMO_ (kcal/mol)	ΔE (kcal/mol)	I (kcal/mol)	A (kcal/mol)	η (kcal/mol)	µ (kcal/mol)	χ (kcal/mol)	ω (kcal/mol)	DM (D)
**1**	−6.227	−3.918	−2.310	6.227	3.918	1.155	−5.073	5.073	11.140	5.6507
**2**	−6.510	−3.989	−2.521	6.510	3.989	1.261	−5.250	5.250	10.930	9.563
**3**	−6.423	−3.980	−2.443	6.423	3.980	1.222	−5.202	5.202	11.076	6.3035
**4**	−6.422	−3.984	−2.438	6.422	3.984	1.219	−5.203	5.203	11.106	6.5282
**5**	−6.503	−3.816	−2.687	6.503	3.816	1.344	−5.159	5.159	9.906	5.8305
**6**	−6.228	−3.869	−2.360	6.228	3.869	1.180	−5.049	5.049	10.801	8.9646
**7**	−6.421	−3.867	−2.554	6.421	3.867	1.277	−5.144	5.144	10.359	6.548
**8**	−6.426	−3.866	−2.560	6.426	3.866	1.280	−5.146	5.146	10.345	6.4041
**9**	−6.499	−4.151	−2.348	6.499	4.151	1.174	−5.325	5.325	12.078	6.7295
**10**	−6.248	−4.183	−2.065	6.248	4.183	1.032	−5.216	5.216	13.176	7.2985
**11**	−6.450	−4.162	−2.288	6.450	4.162	1.144	−5.306	5.306	12.306	4.5204
**12**	−6.450	−4.165	−2.285	6.450	4.165	1.142	−5.307	5.307	12.328	4.6037
**13**	−6.485	−3.786	−2.699	6.485	3.786	1.349	−5.135	5.135	9.772	7.0345
**14**	−6.239	−3.816	−2.424	6.239	3.816	1.212	−5.027	5.027	10.428	8.3989
**15**	−6.428	−3.792	−2.637	6.428	3.792	1.318	−5.110	5.110	9.903	5.0970
**16**	−6.442	−3.793	−2.649	6.442	3.793	1.325	−5.117	5.117	9.885	5.6097

**Table 8:** Vina affinity scoring values (ΔG) (kcal/mol) and pKI for tested compounds.

Compound	Mpro		PLpro		Compound	Mpro		PLpro	
	ΔG	pK_I_	ΔG	pK_I_		ΔG	pK_I_	ΔG	pK_I_
**1**	−8.8	6.46	−8.3	6.09	**11**	−9.3	6.82	−6.4	4.70
**2**	−8.9	6.53	−6.9	5.06	**12**	−9.1	6.68	−7.0	5.14
**3**	−8.6	6.31	−6.5	4.77	**13**	−8.6	6.31	−7.6	5.58
**4**	−7.8	5.72	−6.5	4.77	**14**	−8.6	6.31	−8.2	6.02
**5**	−8.8	6.46	−7.9	5.80	**15**	−8.6	6.31	−6.4	4.70
**6**	−8.9	6.53	−8.1	5.94	**16**	−8.4	6.16	−7.0	5.14
**7**	−8.0	5.87	−6.9	5.06	**Chloroquine**	−5.7	4.18	−5.2	3.82
**8**	−8.5	6.24	−6.4	4.70	**Remdesivir**	−7.4	5.43	−5.7	4.18
**9**	−9.1	6.68	−8.1	5.94	**Amantadine**	−4.5	3.30	−4.1	3.01
**10**	−9.2	6.75	−6.8	4.99					

## Data Availability

Not applicable.

## Supplementary Materials

The following are available online at https://www.mdpi.com/article/10.3390/pharmaceutics13060781/s1, Table S1. The experimental of lipophilicity (logP_TLC_) and hydrophobic index (φ_0_) for compounds **1**–**20**, Table S2. The calculated lipophilicity for compounds **1**–**20**, Table S3. The experimental and predicted logP, Table S4. The local minima of hybrids **1**–**16**, Table S5. Interaction of hybrids **1**, **2**, **6**, **9**, **10**, **11,** and **14** with active site of Mpro and PL protein, Figure S1. The optimized structure of hybrids **1**–**16**, Figure S2. The linear regression between the experimental and literature lipophilicity for standard substance, Figure S3. The similarity analysis of pharmacokinetic parameters of hybrids **1**–**16**, Figure S4. The HOMO and LUMO orbitals for hybrids **1**–**16**, Figure S5. The MEP for hybrids **1**–**16**, Figure S6. Docking pose of COVID-19 Mpro protein complex with hybrids **2** (A), **6** (B), **10** (C), **11** (D), and **14** (E), Figure S7. Docking pose of COVID-19 PLpro protein complex with hybrids **1** (A), **6** (B), **9** (C), and **14** (D).
