# Supplementary material for: Lipophilicity, Pharmacokinetic Properties, and Molecular Docking Study on SARS-CoV-2 Target for Betulin Triazole Derivatives with Attached 1,4-Quinone
Source: Pharmaceutics. 2021 May 23;13(6):781. doi: 10.3390/pharmaceutics13060781 (PMC8224687; doi:10.3390/pharmaceutics13060781)

pharmaceutics

Supplementary Materials: Lipophilicity, Pharmacokinetic Properties, and Molecular Docking Study on SARS-CoV-2 Target for Betulin Triazole Derivatives with Attached 1,4-Quinone

Monika Kadela-Tomanek, Maria Jastrzębska, Krzysztof Marcieniec, Elwira Chrobak [1], Ewa Bębenek and Stanisław Boryczka

Table of Contents

Table S1. The experimental of lipophilicity ($logP_{TLC}$) and hydrophobic index (φ_0) for compounds **1–20**.2
Table S2. The calculated lipophilicity for compounds **1–20**. 2
Table S3. The experimental and predicted logP. 3
Table S4. The local minima of hybrids **1–16**. 3
Table S5. Interaction of hybrids **1**, **2**, **6**, **9**, **10**, **11** and **14** with active site of Mpro and PL protein. 4
Figure S1. The optimized structure of hybrids **1–16**. 6
Figure S2. The linear regression between the experimental and literature lipophilicity for standard substance. 7
Figure S3. The similarity analysis of pharmacokinetic parameters of hybrids **1–16**. 7
Figure S4. The HOMO and LUMO orbitals for hybrids **1–16**. 7
Figure S5. The MEP for hybrids **1–16**. 9
Figure S6. Docking pose of COVID-19 Mpro protein complex with hybrids **2** (A.), **6** (B.), **10** (C.), **11** (D.), and **14** (E.). 10
Figure S7. Docking pose of COVID-19 PLpro protein complex with hybrids **1** (A.), **6** (B.), **9** (C.), and **14** (D.). 12

Table S1. The experimental of lipophilicity ($logP_{TLC}$) and hydrophobic index (φ_0) for compounds **1–20**.

Compound	$logP_{TLC}$	φ_0	Compound	$logP_{TLC}$	φ_0
**1**	4.56	82.40	**11**	5.26	85.94
**2**	4.71	83.14	**12**	5.56	88.32
**3**	5.08	84.76	**13**	4.87	87.13
**4**	5.21	87.00	**14**	4.93	87.76
**5**	4.61	79.81	**15**	5.35	85.11
**6**	4.64	82.83	**16**	5.77	86.18
**7**	5.20	83.48	**17**	5.57	82.62
**8**	5.39	88.21	**18**	5.73	83.67
**9**	4.72	82.43	**19**	6.33	86.06
**10**	4.86	83.85	**20**	6.55	87.13

Table S2. The calculated lipophilicity for compounds **1–20**.

Com.	ALOGPs	AClogP	AlogP	XLOGP2	XLOGP3	milogP	iLOGP	WLOGP	MLOGP	SILICOS-IT
**1**	6.39	6.57	7.75	9.37	10.41	7.88	5.23	8.78	4.19	7.85
**2**	6.58	6.74	7.71	8.79	10.09	7.70	5.28	8.99	4.11	8.44
**3**	6.83	7.06	8.13	10.11	10.99	8.46	5.53	9.36	4.48	8.4
**4**	7.09	7.52	8.80	10.39	11.46	8.67	6.07	9.75	4.64	8.82
**5**	6.26	6.47	7.32	9.28	10.08	8.43	5.56	8.78	4.19	7.85
**6**	6.49	6.63	7.29	8.67	9.76	8.29	5.36	8.99	4.11	8.44
**7**	6.71	6.96	7.70	10.02	10.65	8.80	5.68	9.36	4.48	8.40
**8**	6.98	7.42	8.37	10.27	11.12	8.95	6.35	9.75	4.64	8.82
**9**	6.61	6.99	8.04	9.68	10.81	7.93	5.84	9.09	4.35	8.40
**10**	6.79	7.16	8.00	9.07	10.49	7.75	5.76	9.30	4.27	8.99
**11**	7.02	7.48	8.41	10.42	11.39	8.49	5.90	9.66	4.64	8.95
**12**	7.28	7.94	9.08	10.67	11.86	8.69	6.54	10.05	4.80	9.38
**13**	6.73	7.54	8.48	10.53	11.15	8.96	5.84	9.39	5.12	8.44
**14**	6.89	7.71	8.44	9.92	10.83	8.89	5.72	9.60	5.04	9.02
**15**	7.10	8.03	8.85	11.27	11.72	9.20	6.04	9.96	5.40	8.89
**16**	7.35	8.50	9.52	11.52	12.19	9.30	6.70	10.35	5.56	9.40
**17**	5.38	5.26	6.49	7.81	8.22	7.05	4.89	6.84	5.30	5.84
**18**	5.54	5.43	6.45	7.20	7.91	6.86	4.77	7.04	5.21	6.43
**19**	5.85	5.75	6.87	8.55	8.80	7.75	5.24	7.41	5.59	6.38
**20**	6.17	6.21	7.54	8.80	9.27	8.11	5.74	7.80	5.76	6.80

Table S3. The experimental and predicted logP.

Compound	Experimental $logP_{TLC}$	Predicted logP	Residual
**1**	4.56	4.59	-0.027
**2**	4.71	4.64	0.064
**3**	5.08	5.09	-0.003
**4**	5.21	5.36	-0.156
**5**	4.61	4.59	0.024
**6**	4.64	4.64	0.005
**7**	5.20	5.09	0.113
**8**	5.39	5.36	0.024
**9**	4.72	4.78	-0.060
**10**	4.86	4.83	0.027
**11**	5.26	5.27	-0.016
**12**	5.56	5.55	0.013
**13**	4.87	4.90	-0.034
**14**	4.93	4.95	-0.022
**15**	5.35	5.40	-0.047
**16**	5.77	5.68	0.096

Table S4. The local minima of hybrids **1–16**.

Compound	First area	Second area	Third area	Fourth area
**1**	−2.007	−2.721; −1.905	−2.231; −3.102; −2.612; −1.088	−2.993
**2**	−1.905	−2.721; −2.231	−2.286; −3.156; −2.612; −1.361	−2.612
**3**	−1.905	−2.721; −2.231	−2.395; −3.156; −2.667; −1.361	−1.905; −2.395
**4**	−1.905	−2.721; −2.231	−2.395; −3.156; −2.721; −1.361	−1.905; −2.503
**5**	−1.796	−2.612; −1.796	−2.068; −2.993; −2.503; −1.143	−2.721
**6**	−1.796	−2.503; −1.361	−2.286; −2.993; −2.612; −1.197	−2.612
**7**	−1.796	−2.612; −1.361	−2.286; −3.102; −2.612; −1.197	−1.905; −2.503
**8**	−1.796	−2.612; −1.361	−2.286; −3.102; −2.612; −1.252	−1.905; −2.503
**9**	−2.007	−2.721; −1.905	−2.231; −3.102; −2.612; −1.088	−2.993
**10**	−1.905	−2.721; −2.231	−2.286; −3.102; −2.612; −1.088	−2.612
**11**	−1.959	−2.721; −2.231	−2.449; −3.156; −2.721; −1.361	−1.905; −2.503
**12**	−1.959	−2.721; −2.231	−2.449; −3.156; −2.721; −1.361	−1.905; −2.449
**13**	−2.077	−2.068	−2.340; −3.102; −2.667; −1.252	−2.993
**14**	−2.068	−1.469	−2.340; −3.102; −2.667; −1.252	−2.667
**15**	−2.068	−1.633	−2.395; −3.102; −2.667; −1.252	−1.905; −2.340
**16**	−2.068	−1.469	−2.395; −3.102; −2.667; −1.361	−1.905; −2.503

Table S5. Interaction of hybrids **1**, **2**, **6**, **9**, **10**, **11** and **14** with active site of Mpro and PL protein.

Protein.		Ligand		Interaction	
**Name**	**Residue**	**Name**	**Residue**	**Type**	**Distance (Å)**
Mpro	Gly143	**2**	carbonyl oxygen at C-8q	Conventional hydrogen bond	2.30
	His41		chlorine atom	Carbon hydrogen bond	2.97
	Asn142		carbonyl oxygen at C-8q	Carbon hydrogen bond	2.93
	Asn142		quinoline nitrogen	Carbon hydrogen bond	2.71
	Cys145		pyridine ring	π-sulfur	3.62
	His41		pyridine ring	π-π stacked	4.74
	His41		benzoquinone ring	π-π stacked	5.73
	Cys145		benzoquinone ring	π-alkyl	4.46
	Pro168		C-29	Alkyl-alkyl	3.62
	Pro168		C-30	Alkyl-alkyl	4.49

	Pro168		C-12	Alkyl-alkyl	4.77
	Pro168	**6**	quinoline nitrogen	Carbon hydrogen bond	3.08
	Asn142		triazole ring	Carbon hydrogen bond	3.24
	Glu166		triazole ring	π-anion	3.23
	Leu141		triazole ring	Amide-π stacked	4.59
	Pro168		pyridine ring	π-alkyl	4.96
	Met49		C-30	Alkyl-alkyl	5.20
	Met165		C-30	Alkyl-alkyl	5.44
	Met49		C-12	Alkyl-alkyl	3.71
	Met49		C-29	Alkyl-alkyl	4.44
	Met165		C-22	Alkyl-alkyl	4.83
	His41		C-30	π-alkyl	4.03
	Gly143	**10**	carbonyl oxygen at C-8q	Conventional hydrogen bond	2.17
	His41		chlorine atom	Carbon hydrogen bond	3.02
	Asn142		carbonyl oxygen at C-8q	Carbon hydrogen bond	2.96
	Asn142		quinoline nitrogen	Carbon hydrogen bond	2.59
	Cys145		pyridine ring	π-sulfur	3.55
	His41		pyridine ring	π-π stacked	4.80
	His41		benzoquinone ring	π-π stacked	5.66
	Cys145		benzoquinone ring	π-alkyl	4.42
	Pro168		C-29	Alkyl-alkyl	4.87
	Pro168		C-30	Alkyl-alkyl	4.78
	Pro168		C-12	Alkyl-alkyl	4.81
	Asn142	**11**	C-28	Carbon hydrogen bond	3.49
	Glu166		triazole ring	π-anion	3.01
	Leu167		pyridine ring	Amide-π stacked	4.28
	Leu167		benzoquinone ring	Amide-π stacked	4.81
	Pro168		pyridine ring	π-Alkyl	3.96
	Pro168		benzoquinone ring	π-Alkyl	4.20
	Met49		C-30	Alkyl-alkyl	5.37
	Met49		C-12	Alkyl-alkyl	4.13
	Met165		C-29	Alkyl-alkyl	5.27
	Met49		C-29	Alkyl-alkyl	3.62
	His41		C-29	π-Alkyl	3.85
	Gln189	**14**	triazole ring	π-sigma	2.45
	His41		pyridine ring	π-π-stacked	4.53
	His41		benzoquinone ring	π-π stacked	5.36
	Met165		triazole ring	π-Alkyl	4.80
	Cys145		pyridine ring	π-Alkyl	4.81
	Cys145		benzoquinone ring	π-Alkyl	4.98
	Pro168		C-21	Alkyl-alkyl	5.11
PLpro	Thr301	**1**	betulin hydroxyl group	Conventional hydrogen bond	2.92
	Asn267		carbonyl oxygen at C-8q	Carbon hydrogen bond	2.63
	Tyr268		triazole ring	π-sigma	2.62
	Tyr268		triazole ring	π-π T-shaped	5.42
	Pro248		pyridine ring	Alkyl-alkyl	4.22
	Pro248		benzoquinone ring	Alkyl-alkyl	4.27
	Pro248		C-24	π-alkyl	4.11
	Pro248		C-23	π-alkyl	4.21
	Leu162		C-16	Alkyl-alkyl	4.78
	Arg166	**6**	betulin carbonyl group	Conventional hydrogen bond	2.16
	Tyr268		triazole ring	π-π T-shaped	5.38

Pro247		benzoquinone ring	Amide-π stacked	5.56
Pro248		pyridine ring	Alkyl-alkyl	4.40
Pro248		benzoquinone ring	Alkyl-alkyl	4.47
Pro248		C-24	π-alkyl	4.13
Pro248		C-23	π-alkyl	3.80
Leu162		C-16	Alkyl-alkyl	4.63
Leu162		C-22	Alkyl-alkyl	5.16
Arg166		betulin hydroxyl group	Conventional hydrogen bond	2.20
Tyr268		triazole ring	Carbon hydrogen bond	2.10
Tyr268		triazole ring	π-π T-shaped	4.96
Pro248		pyridine ring	π-alkyl	4.55
Pro248	**9**	benzoquinone ring	π-alkyl	5.14
Pro248		C-24	Alkyl-alkyl	4.42
Pro248		C-23	Alkyl-alkyl	4.54
Leu162		C-21	Alkyl-alkyl	5.22
Leu162		C-16	Alkyl-alkyl	4.77
Lys157		carbonyl oxygen at C-5q	Conventional hydrogen bond	2.17
Lys157		oxygen at C-7q	Conventional hydrogen bond	2.32
Gly163		carbonyl oxygen at C-5q	Carbon hydrogen bond	2.82
Leu162		benzene ring	π-alkyl	4.75
Leu162		benzoquinone ring	π-alkyl	4.07
Pro248	**14**	C-26	Alkyl-alkyl	4.33
Pro248		C-12	Alkyl-alkyl	4.99
Pro248		C-6	Alkyl-alkyl	3.81
Met208		C-30	Alkyl-alkyl	5.46
Tyr264		C-26	π-alkyl	5.29
Tyr264		C-16	π-alkyl	4.08

Figure S1. The optimized structure of hybrids **1-16**.

**Comp.**	Structure	**Comp.**	Structure
**1**	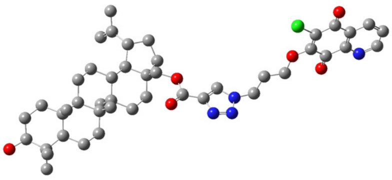	**9**	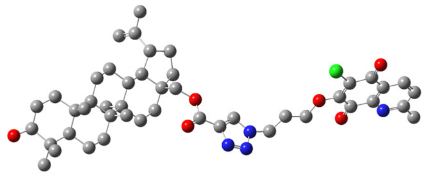
**2**	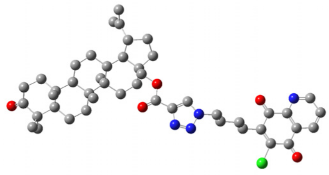	**10**	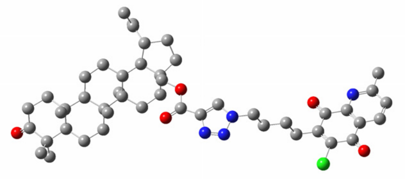
**3**	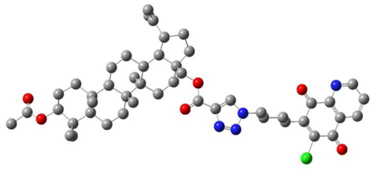	**11**	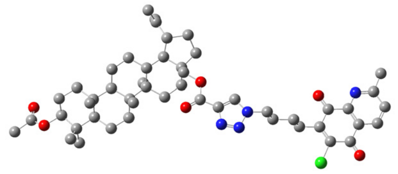
**4**	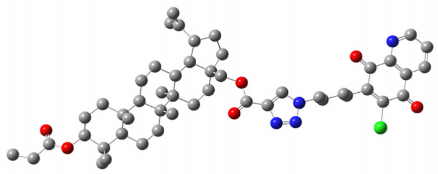	**12**	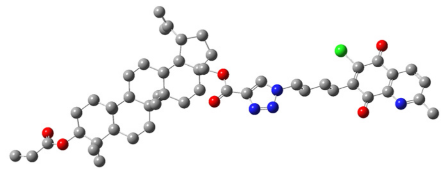

5

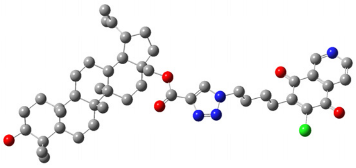

13

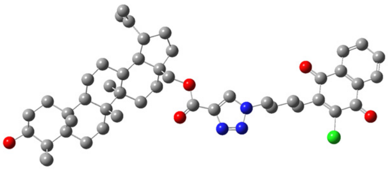

6

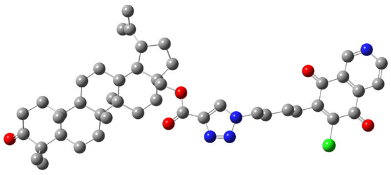

14

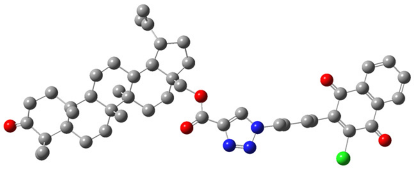

7

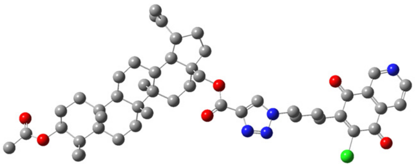

15

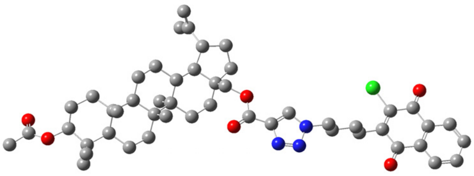

8

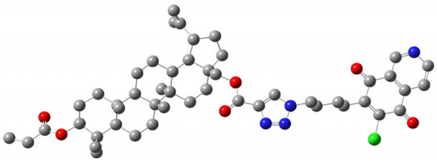

16

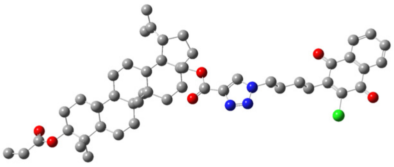

Figure S2. The linear regression between the experimental and literature lipophilicity for standard substance.

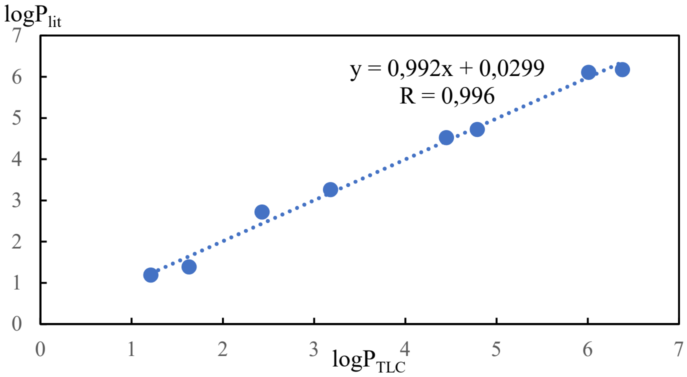

Figure S3. The similarity analysis of pharmacokinetic parameters of hybrids **1–16**.

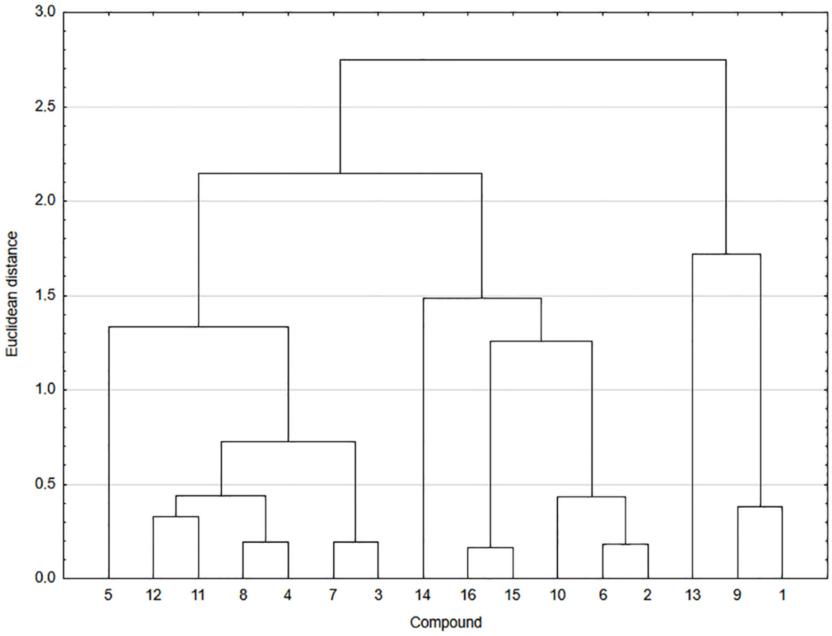

Figure S4. The HOMO and LUMO orbitals for hybrids **1–16**.

**Compound.**	HOMO and LUMO orbitals	**Compound.**	HOMO and LUMO orbitals
**1**	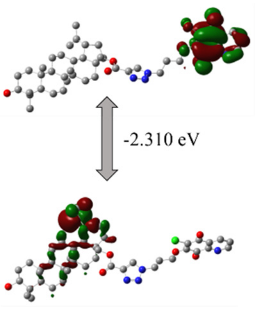	**9**	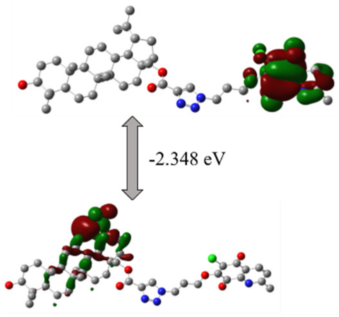
**2**	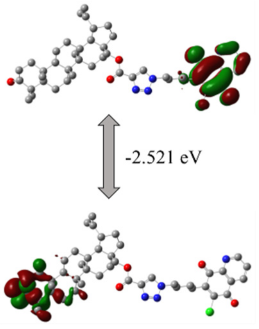	**10**	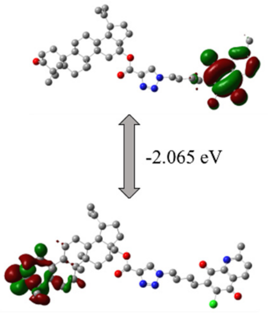
**3**		**11**	

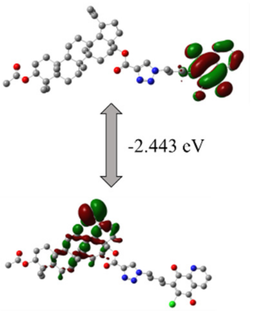

4

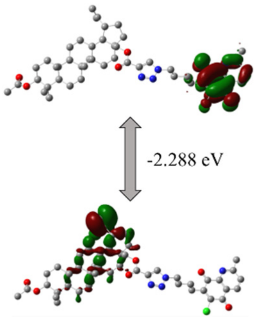

12

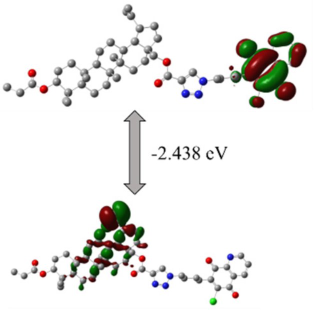

5

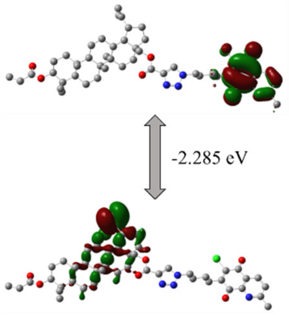

13

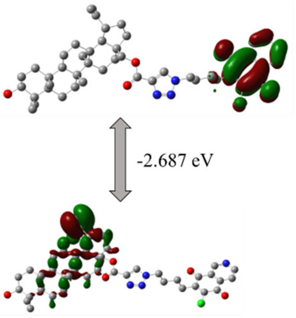

6

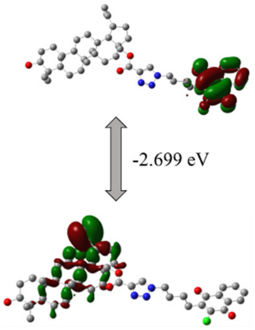

14

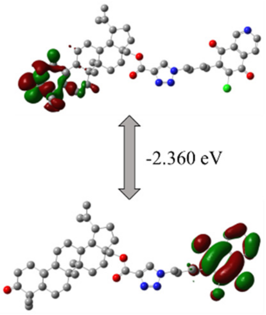

7

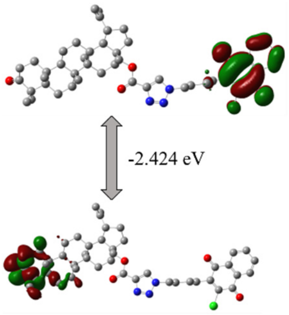

15

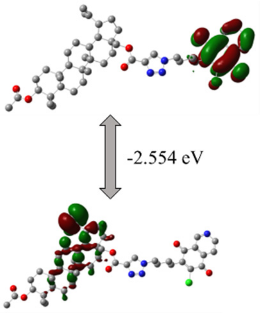

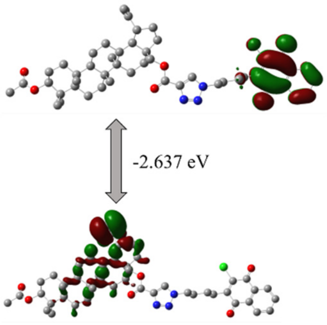

**8**	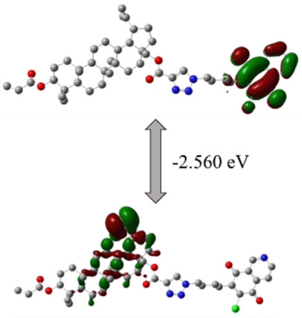	**16**	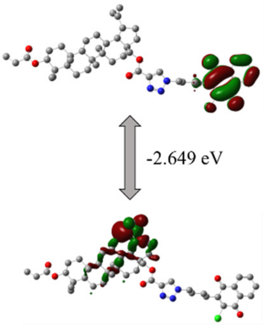

Figure S5. The MEP for hybrids **1–16**.

**Com-pound.**	MEP	Com-pound	MEP
**2**	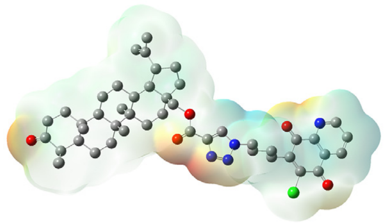	**10**	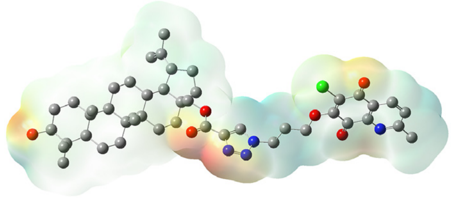
**3**	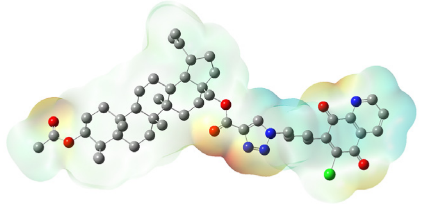	**11**	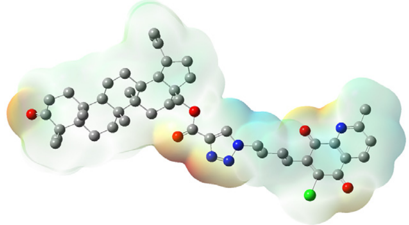
**4**	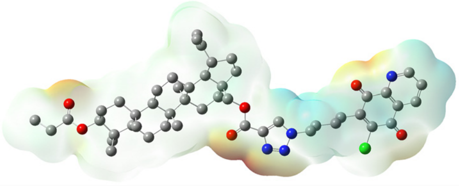	**12**	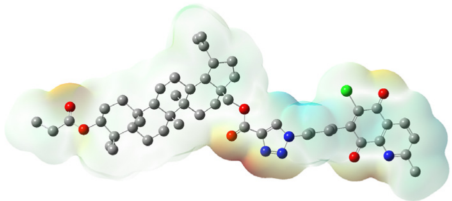
**6**	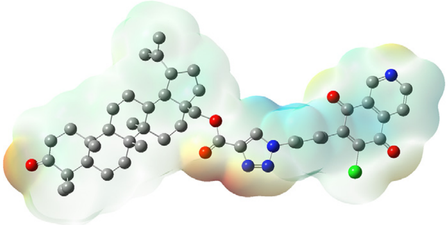	**14**	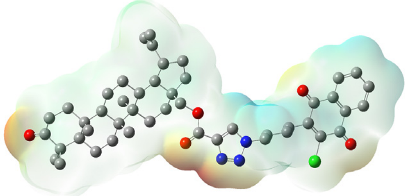

7

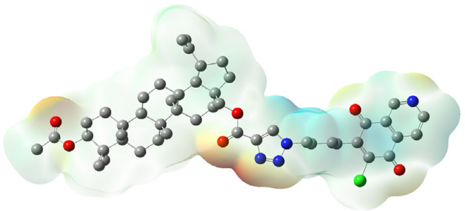

15

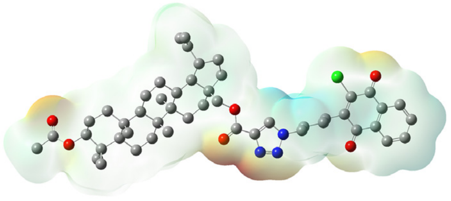

8

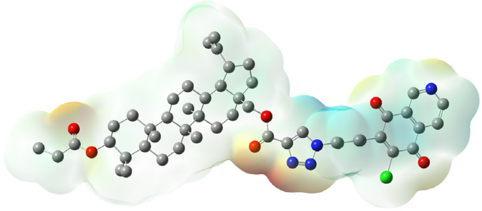

16

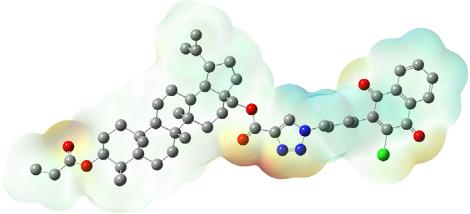

Figure S6. Docking pose of COVID-19 Mpro protein complex with hybrids **2** (A.), **6** (B.), **10** (C.), **11** (D.), and **14** (E.).

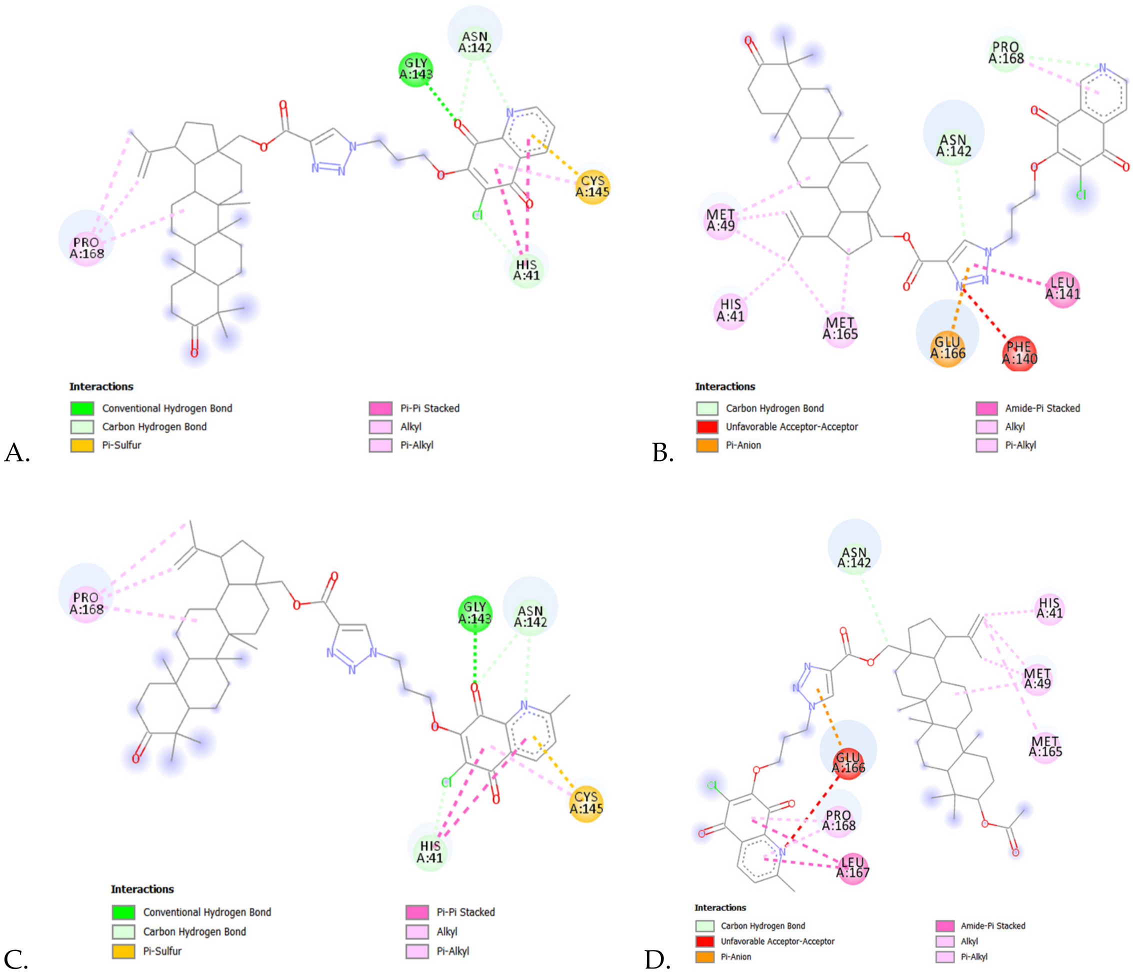

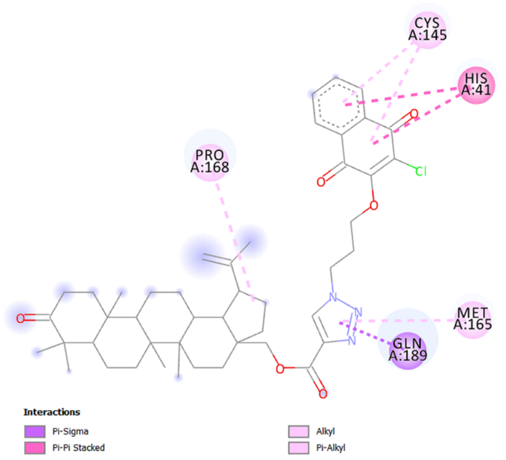

E.

Figure S7. Docking pose of COVID-19 PLpro protein complex with hybrids **1** (A.), **6** (B.), **9** (C.), and **14** (D.).

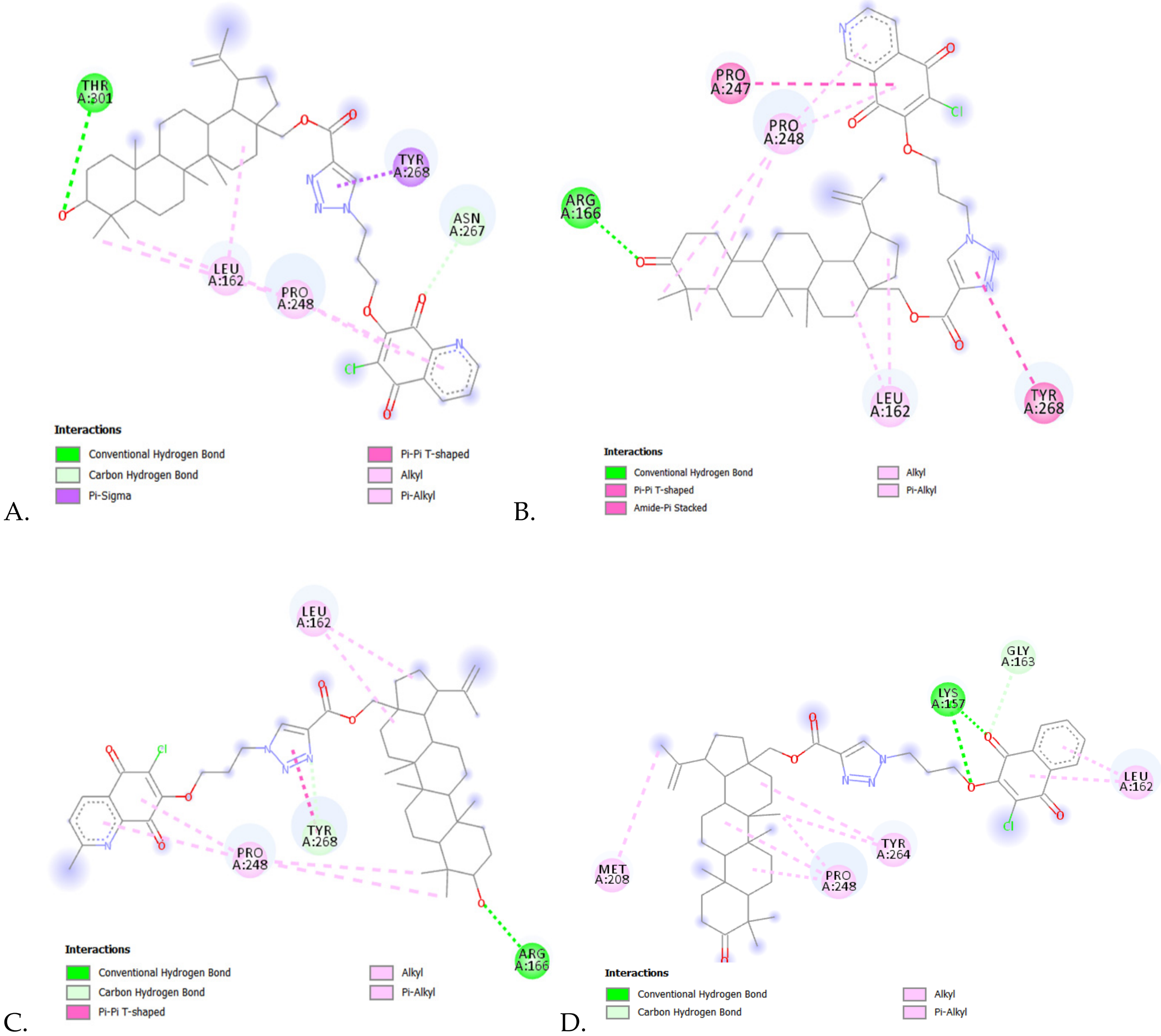

Supplement: Supplementary file 1 [file pharmaceutics-13-00781-s001.zip › pharmaceutics-1231363-supplementary.pdf]
